# Determinants of Anti-S Immune Response at 9 Months after COVID-19 Vaccination in a Multicentric European Cohort of Healthcare Workers—ORCHESTRA Project

**DOI:** 10.3390/v14122657

**Published:** 2022-11-28

**Authors:** Giulia Collatuzzo, Vittorio Lodi, Daniela Feola, Giuseppe De Palma, Emanuele Sansone, Emma Sala, Christian Janke, Noemi Castelletti, Stefano Porru, Gianluca Spiteri, Maria Grazia Lourdes Monaco, Francesca Larese Filon, Corrado Negro, Luca Cegolon, Jana Beresova, Eleonora Fabianova, Lucia A. Carrasco-Ribelles, Pere Toràn-Monserrat, Marta Maria Rodriguez-Suarez, Guillermo Fernandez-Tardon, Shuffield S. Asafo, Giorgia Ditano, Mahsa Abedini, Paolo Boffetta

**Affiliations:** 1 Department of Medical and Surgical Sciences, University of Bologna, 40138 Bologna, Italy; 2 IRCCS Azienda Ospedaliero-Universitaria di Bologna, 40138 Bologna, Italy; 3 Department of Medical and Surgical Specialties, Radiological Sciences and Public Health, University of Brescia, 25121 Brescia, Italy; 4 Division of Infectious Diseases and Tropical Medicine, LMU Klinikum, 80331 Munich, Germany; 5 Section of Occupational Medicine, Department of Diagnostics and Public Health, University of Verona, 37134 Verona, Italy; 6 Clinical Unit of Occupational Medicine, University Hospital of Verona, 37134 Verona, Italy; 7 Unit of Occupational Medicine, University of Trieste, 34121 Trieste, Italy; 8 Epidemiology Department, Regional Authority of Public Health, 97401 Banská Bystrica, Slovakia; 9 Occupational Health Department, Regional Authority of Public Health, 97401 Banská Bystrica, Slovakia; 10 Unitat de Suport a la Recerca Metropolitana Nord, Institut Universitari d’Investigació en Atenció Primària Jordi Gol, 08302 Mataró, Spain; 11 Germans Trias i Pujol Research Institute, 08911 Badalona, Spain; 12 Department of Medicine, Faculty of Medicine, Universitat de Girona, 17001 Girona, Spain; 13 Multidisciplinary Research Group in Health and Society, 08001 Barcelona, Spain; 14 Health Research Institute of Asturias, Centro de Investigación Biomédica en Red en Epidemiología y Salud Pública (CIBERESP), 33001 Oviedo, Spain; 15 Public Health Department, University of Oviedo, 33001 Oviedo, Spain; 16 Stony Brook Cancer Center, Stony Brook University, Stony Brook, NY 10041, USA

**Keywords:** COVID-19, vaccine, serology, antibody level, immunization, temporal trends

## Abstract

**Background:** The persistence of antibody levels after COVID-19 vaccination has public health relevance. We analyzed the determinants of quantitative serology at 9 months after vaccination in a multicenter cohort. **Methods:** We analyzed data on anti-SARS-CoV-2 spike antibody levels at 9 months from the first dose of vaccinated HCW from eight centers in Italy, Germany, Spain, Romania and Slovakia. Serological levels were log-transformed to account for the skewness of the distribution and normalized by dividing them by center-specific standard errors. We fitted center-specific multivariate regression models to estimate the cohort-specific relative risks (RR) of an increase of one standard deviation of log antibody level and the corresponding 95% confidence interval (CI), and combined them in random-effects meta-analyses. Finally, we conducted a trend analysis of 1 to 7 months’ serology within one cohort. **Results**: We included 20,216 HCW with up to two vaccine doses and showed that high antibody levels were associated with female sex (*p* = 0.01), age (RR = 0.87, 95% CI = 0.86–0.88 per 10-year increase), 10-day increase in time since last vaccine (RR = 0.97, 95% CI 0.97–0.98), previous infection (3.03, 95% CI = 2.92–3.13), two vaccine doses (RR = 1.22, 95% CI = 1.09–1.36), use of Spikevax (OR = 1.51, 95% CI = 1.39–1.64), Vaxzevria (OR = 0.57, 95% CI = 0.44–0.73) or heterologous vaccination (OR = 1.33, 95% CI = 1.12–1.57), compared to Comirnaty. The trend in the Bologna cohort, based on 3979 measurements, showed a decrease in mean standardized antibody level from 8.17 to 7.06 (1–7 months, *p* for trend 0.005). **Conclusions:** Our findings corroborate current knowledge on the determinants of COVID-19 vaccine-induced immunity and declining trend with time.

## 1. Introduction

Health care workers (HCW) were the first to face COVID-19 infection, and still remain a particularly exposed population [1,2]. Consequently, they were also among the first population groups to be recommended for vaccination once it was introduced in Europe [3].

Several studies supported the safety of the new developed vaccines, including those based on m-RNA (Comirnaty (BioNTech/Pfizer) [4] and Spikevax (Moderna) [5]). Data also showed that vaccines were effective against the development of symptoms and reduced the risk of infection, and led to a reduction of deaths and hospitalization as well [6,7,8,9]. The persistence of specific antibodies is considered a marker of capability of the immune system to protect against a given infection [10]. The amount of antibodies against the targeted microorganism depends on the type of vaccine and the type of induced antibodies, where neutralizing antibodies represent those conferring protection [11].

To assess the potential responsiveness of an individual humoral response to COVID-19 infection, specific antibody testing is reliable, as its positivity correlates with neutralizing antibodies. According to previous literature, anti-S1 (*Euroimmun*, *ELISA*, *anti-S1*) results showed a good correlation with neutralizing antibodies, even better than the correlation found with anti-NP (*Abbott*, *ELISA*, *anti-NP*) [12]. Infection was less likely in subjects with anti-S antibodies, in which, in addition, no symptomatic infections were detected in workers [13].

Anti-N and anti-S antibodies detected with the electrochemiluminescence immunoassay (ECLIA) method have been reported to persist at 9 months in ≥ 90% of subjects who were infected by COVID-19 [12].

To date, the persistence of immunity after COVID-19 vaccination has not been clearly defined, as well as the clinical relevance of different antibody levels.

This depends, among the other factors, on the paucity of data collected on a large scale at certain times after vaccination.

Thus, current evidence is insufficient to (i) indicate which antibody titers confer protection against COVID-19; (ii) if a booster dose is not required for previously infected subjects; (iii) which is the best time schedule for a booster dose in never-infected subjects and, if needed, in previously infected ones. Accurate knowledge of these aspects would be translated into public health guidelines to be applied both in the hospital and in the general population.

In addition, it would bring new understanding on the occurrence of breakthrough infections, which have been increasingly reported following large-scale vaccination campaigns worldwide.

Our previous analysis of the ORCHESTRA project, a collaboration of cohorts of HCW and other populations from multiple countries addressing various aspects of the COVID-19 pandemic, showed a decrease of anti-SARS-CoV-2 IgG titers at 6 months after the second dose [14]. In the present study we aimed to expand the analysis of COVID-19 antibody titers to 9 months after the first vaccine dose. In particular, we aimed at identifying the predictors of immune response, by exploring HCW-related and vaccination-related characteristics.

## 2. Methods

ORCHESTRA comprises a prospective multicenter cohort including more than 60,000 HCW employed in hospitals in different European countries [14]. This analysis involves HCW from one center in Germany (Munich), four centers in Italy (Bologna, Brescia, Trieste and Verona), two centers from Spain (Northern Barcelona region and Oviedo) and several centers in Slovakia (the latter treated as an individual cohort), with serological results at 9 months after first vaccination dose. Data on sociodemographic characteristics, results of PCR testing and vaccination status, including date of vaccination doses and type, were either abstracted from medical surveillance records or collected using questionnaires or ongoing loco-regional databases. Results on level of anti-S antibodies were either collected from medical records or generated through ad-hoc testing.

Results on level of anti-N antibodies were obtained from 10,121 subjects from 3 cohorts (Brescia, Munich and Barcelona).

All cohorts included in the ORCHESTRA project have undergone extensive data harmonization.

The proportion of HCW who did not develop a serological response after vaccination varied across the cohorts from 0% to 1.1%; these subjects were excluded from all analysis on serological results, as in previous published analyses [15,16]. The present study comprises 20,476 HCW with available and positive serology results during a 9-month timeframe from first dose administration, defined as an interval of 210–330 days.

The primary outcome of this analysis was the level of serum anti-S antibodies at 9 months. Methods of measurement of antibody levels varied across centers and time periods; details are reported in Supplementary Table S1.

For quantitative analyses, anti-S antibody levels were log-transformed to account for the skewness of the distribution. To take into account the heterogeneity in analytical methods, log-transformed results were normalized by dividing them by the center-specific standard errors. This approach was used also in previous analyses within the ORCHESTRA project [15,16], in order to obtain measurements that were comparable among the cohorts even if different types of tests were used.

We fitted multivariate linear regression models to estimate—for each determinant included—relative risks (RR) and corresponding 95% confidence intervals (CI) of an increase of one standard deviation (SD) of normalized log-transformed antibody level. The following covariates were considered: sex, age, time since last vaccine dose, previous COVID-19 infection, number of vaccine doses (one or two) and type of vaccine (Comirnaty, Spikevax, Vaxzevria, or mixed vaccines). Previous COVID-19 infection was assessed using results on anti-N antibody tests (dichotomous variable, N = 10,105), as well as based on results of PCR (never, before first vaccine dose, after first vaccine dose, both before and after first vaccine dose; N = 20,208).

In a secondary analysis, we included also job title in the regression model (not available for the Munich cohort). In addition, we stratified the analysis according to type of anti-S serologic test: RBD-based cheminulinescence immunoassay (CLIA) (cohorts from Munich, Bologna and Trieste) vs. other CLIA or ELISA (other cohorts).

Moreover, we performed an additional analysis where we excluded cohorts whose standardized antibody levels appeared to be different from the others.

Finally, we analyzed the standardized results of serologic tests in the Bologna cohort between month 1 and month 7 after the first vaccine dose. This was the only cohort with measurements of more than 40 HCW on each month during this interval. Because of the association between age and antibody level, we adjusted by age using the standard European population.

We used the Stata^®^ software V. 17 (StataCorp LP, College Station, TX, USA) in the statistical analysis.

The study was approved by the Italian Medicine Agency (AIFA) and the Ethics Committee of Italian National Institute of Infectious Diseases (INMI) Lazzaro Spallanzani. Each cohort was approved by the local ethical board.

## 3. Results

Overall, 20,476 vaccinated HCW had available blood samples at 9 months since first vaccine dose. Table 1 reports the distribution of standardized anti-S serology results by sociodemographic and vaccine-related characteristics, and Table 2 summarizes the results on time intervals between vaccine, first serology test, and 9-month serology tests. Most subjects were from the cohorts from Brescia (6250, 30.5%), Bologna (4402, 21.5%), Munich (3473, 17.0%) and Verona (3250, 15.9%). The study population was mostly constituted of women (72.8%) and people aged 50 or older (41.2%). Nurses (37.7%) and physicians (26.0%) represented the largest occupational groups. Up to 87.4% of the individuals had never been infected by COVID-19 at the time of the serology sample, while 10.1% were infected with COVID-19 before vaccination and 2.5% resulted to be infected after the first dose or at both times, based on PCR tests. When considering anti-N seropositivity as diagnostic method for COVID-19 infection, the proportion of ever infected individuals increased to 17.9%, based on 10,121 subjects. Last, most HCW received a Comirnaty vaccine (97.1%) and completed the two-dose cycle (98.7%).

The results of the multiple linear regression conducted on 20,216 HCW with available information are shown in Table 3. Women were more likely to have a higher anti-S serology level than men (*p* = 0.01). Age was inversely related to serological level at 9 months, with an RR = 0.87 (95% CI = 0.86–0.88) for a 10-year increase. The RR for a 10-day increase in time since last vaccine dose was 0.97 (95% CI 0.97–0.98). We distinguished the analyses of COVID-19 infection by type of test, separating PCR (20,208 observations) and anti-N (10,105 observations). In the PCR-based analysis, subjects previously infected had markedly higher anti-S antibody levels than those never infected, with small differences by timing of the infection in relation to COVID-19 vaccination (OR = 2.64, 95% CI = 2.53–2.76 for being infected before vaccination; OR = 2.68, 95% CI = 2.47–2.92 for infected after the first dose; OR = 2.87, 95% CI = 2.19–3.77 for being infected both times). In the analysis based on anti-N, we detected a 4-fold increased OR (OR = 4.02, 95% CI = 3.86–4.19) of increased antibody level in HCW being anti-N positive compared to those who always tested negative. Moreover, HCW with two vaccine doses had a 22% higher likelihood to have higher anti-S antibody titers at 9 months compared to HCW with one dose (*p* < 0.001). Last, when comparing the anti-S serologies of subjects receiving different types of vaccines, Spikevax and heterologous vaccination conferred a higher anti-S serological response at 9 months compared to Comirnaty (OR = 1.51, 95% CI = 1.39–1.64 and OR = 1.33, 95% CI = 1.12–1.57 respectively) while use of Vaxzevria was associated with a lower antibody level at 9 months (OR = 0.57, 95% CI = 0.44–0.73).

When we included job title in the regression model shown in Table 3, the sample size was reduced to 16,724 subjects. Job title did not have an association with anti-S antibody level, while the results for the other variables did not change compared to the main analysis (Supplementary Table S2).

We repeated the main analysis after excluding the cohort from Munich, which showed the lowest standardized level of anti-S antibodies, obtaining results which were comparable to those reported in Table 2.

The results of the analysis stratified by type of anti-S serological assay are reported in Supplementary Table S3. RBD-based CLIA assays were used in the cohorts from Munich, Bologna and Trieste (N = 9623), while non-RBD-CLIA or other ELISA tests were used in the other cohorts (N = 10,593). Results of the regression analysis were consistent between the two groups of cohorts.

**Figure 1:**
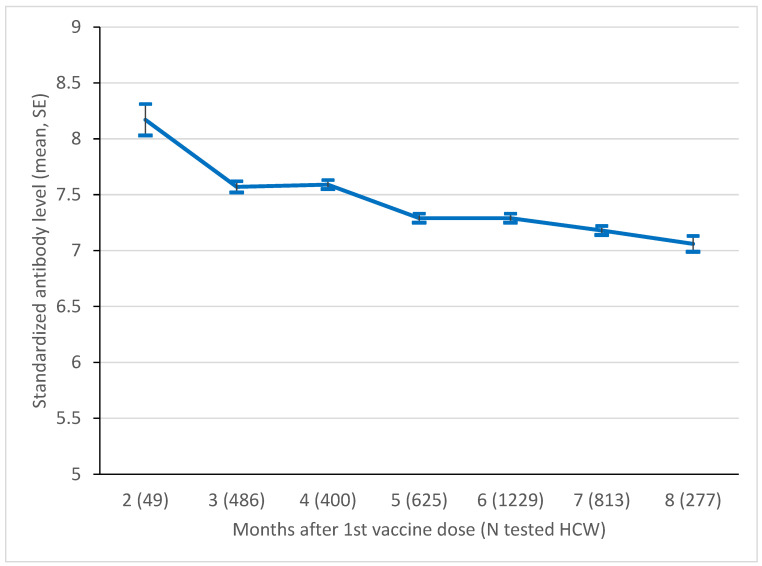
Mean level of standardized log anti-S level in the Bologna cohort, by month since 1st vaccine dose. SE, standard error. *p*-value of test for linear trend = 0.005.

The results of the age-adjusted standardized results serologic tests in the cohort from Bologna between month 1 and month 7 after the first vaccine dose, based on a total of 3979 measurements, are reported in Figure 1. The mean standardized level decreased from 8.17 (SE 0.14) at month 1 to 7.06 (SE 0.07) at month 7 (p for linear trend 0.005).

## 4. Discussion

This analysis pooled the results of COVID-19 antibodies at 9 months from first vaccination in about 20,500 vaccinated HCW from eight different European cohorts included in the ORCHESTRA project.

We identified the following predictors of higher anti-S serological levels: female sex, previous COVID-19 infection, two vaccination doses, use of Spikevax vaccine and heterologous vaccination. Older age and time from first vaccination were inversely related to antibody level, as well as Vaxzevria vaccine. Additionally, our results are consistent when considering RDB-based CLIA and other CLIA/ELISA tests. A mild decrease in antibody level was observed from first to seventh month in the Bologna cohort, with an overall persistence of vaccine-induced immunization. These results are consistent with previous findings from the ORCHESTRA study, including a partially overlapping set of cohorts [15,16], and corroborate previous literature on the topic [17,18,19,20].

Our data refer to the anti-S serologies collected at 9 months after vaccination in a population of HCW, most of whom completed the two-dose scheduled vaccination for COVID-19. Noticeably, most of the participants were from Italy, which was highly affected by the pandemic. In particular, the data we collected refer to the period before the Omicron variant of SARS-CoV-2 became prevalent in these populations.

Anti-N and anti-S antibodies detected with the ECLIA method have been reported to persist at 9 months in ≥ 90% of subjects who were infected by COVID-19 [11]. Additionally, a recent study conducted in Spain (the ProHEpiC-19 cohort study) demonstrated the persistence of positive anti-N antibodies until 450 days (15 months) [21].

The persistence of vaccine-induced serology has not been clearly defined, mainly because of insufficient data collected on a large scale. When looking at the quantitative serology trend in the eight previous months, available for the Bologna cohort, we observed a persistent and quite constant level of antibodies in both sexes (Figure 1). This result is interesting, considering the homogeneous use of the ECLIA test, and the quite homogeneous and relatively high number of serology samples available by month, which allowed to present solid data supporting the duration of COVID-19 vaccine-induced antibodies in time. These results are consistent with a previous Polish study, where 8-month antibodies were detected in 100 hospital employees after two doses of the Comirnaty vaccine [22]. A subsequent study measured the level of antibodies in the same population at 10 months, detecting a small but significant decline [23]. In particular, the authors found that antibodies decreased by 13% in subjects without a history of infection, and by 21% in recovered COVID-19 patients. The authors also noted that around 70% of the recovered HCW, but none of the uninfected, had an antibody level that was higher than 1700 binding antibody units/mL detected with ELISA, which is the threshold reported to confer full protection [24].

The progressive decline in vaccine immunity is common and widely known [25]. Among the factors that influence the immune response to vaccination, which varies substantially between the individuals, there are intrinsic host factors (such as age, sex, genetics and comorbidities), perinatal factors (such as birth weight, feeding method and maternal factors), and extrinsic factors (such as preexisting immunity, microbiota and antibiotics) [25].

In this and in our previous studies [15,16], we explored in particular the vaccine characteristics associated with qualitative and quantitative serology at a certain time. The overwhelming majority of cohort members were administered with either Comirnaty or Spikevax. Both are m-RNA vaccines, representing a novel approach based on their mechanism. Our results are consistent with several studies that described a longer persistency of antibodies after Spikevax vaccination than Comirnaty, as well as after heterologous than homologous vaccination. In fact, heterologous prime-boost has been shown to be unexpectedly more effective than homologous vaccination [26,27].

The analysis performed within the Bologna cohort showed a decline of about one SE of the log-transformed antibody level during 7 months from first vaccination. Despite not being an individual-level analysis, to our knowledge this is the largest dataset in which the time trend of COVID-19 vaccine-induced immunization has been studied. It would be interesting to extend in time the investigation of quantitative trends in antibody level, and to compare them between different populations (e.g., general population and HCW). In fact, as HCW are particularly exposed to COVID-19 during working activity, it would be interesting to disentangle the effect exerted by the potential subclinical contact with the virus from that exerted by vaccination.

A recent review described the kinetics of the COVID-19 vaccine-induced immune response [17]. The authors mentioned how antibody titers were found to decline to 64% of the initial level at 9 months from infection in Wuhan patients, and were decreased by 70% compared to 1-month serology after 1 year [17]. Neutralizing antibody levels were also reported to decline with time, together with their protective effect, but without a well-assessed relationship between the quantitative reduction and the decrease in their protection. Moreover, higher neutralizing antibody titers were measured in individuals with previous COVID-19 infection, being specifically associated with disease severity [17].

About 2.5% of the study participants got infected after the first dose according to PCR tests. According to our results, and consistently with a large body of literature [15,16,17,18,19], previous infection enhanced the likelihood of high levels of antibodies against COVID-19 in vaccinated subjects, which, in detail, we found to be particularly high in HCW who had more recent infections (i.e., breakthrough infection (BI) HCW) and who had multiple exposures to the virus (i.e., HCW who got infected both before the first dose and between the first and the second dose).

The proportions of previously infected HCW differed when considering PCR tests and anti-N tests (in particular, Roche Elecsys-Elecsys^®^ Anti-SARS-CoV-2-used in the Munich and Brescia cohorts; Nucleocapside Protein IgM and IgG ELISA kits-Immunodiagnostic Limited©-used in the Northern Barcelona region). This is consistent with the description of asymptomatic infections, which remain undiagnosed at the time of their occurrence [28]. On the other hand, PCR tests have higher sensitivity and specificity than anti-N [28,29], which can lead to false positive results [30]. As overall there may be a certain underestimation of the number of infections, a consequence is the overrated attribution of immunization to vaccination. Indeed, it is important to take into account the different potential reading of the results we show, based on the percentages of positive tests before the vaccine. In any case, the literature states the importance of vaccination in both never and ever infected, these latest having an enhanced level of antibodies through the vaccination.

We investigated whether the type of test influenced the identification of the determinants of serological level. When comparing the results by type of test, we found no significant difference in the determinants of antibody levels, suggesting the same effect of the characteristics analyzed in relation to RBD-based CLIA and other tests. Thus, the determinants of serological response appear to be similar irrespectively of the test used.

The main strength of the present study lies in the prospective design, matched with a large sample size resulting from pooling of eight cohorts of HCW from four different European countries. Most of the HCW received two doses of vaccine, and thus our results refer to the effect of a full COVID-19 vaccine schedule, excluding the booster (third) dose. Moreover, most of the cohorts come from Italy, which was intensely hit by the COVID-19 pandemic.

Regarding the antibody measurements, the heterogeneity in blood tests among the cohorts was overcome by standardizing the antibody levels, in order to obtain comparable results. In this way, we were able to identify actual differences in antibody levels among cohorts according to various characteristics, and predictors of high immunological response 9 months after first vaccination. This approach has been reported as successful in our previous studies, and could represent a useful method to address test heterogeneity in order to compare studies conducted in different populations.

Another strength point is the presentation of monthly quantitative serologies collected within the Bologna cohort, which represents one of the few available descriptions of this information and the largest one provided to date, despite not offering individual-level data.

The overall consistency of our results with previous literature supports the robustness of the analysis. Moreover, internal validity was checked through several sensitivity analyses, allowed by the large number of subjects included in the analysis. Finally, this study was part of a series of periodic updates on the serology data of vaccinated HCW from the ORCHESTRA pooled study, which has already produced results at 3 and 6 months after vaccination [15,16]. This project will provide further results as follow-up of vaccinated HCW continues into the future, including individual-level trends in antibody levels.

A limitation of this study is the lack of information on HCW’s health status, which could have acted as a confounder. Similarly, lifestyle factors (e.g., smoking, dietary habits) [25], which may influence the development of vaccine-induced immune responses against COVID-19, were not taken into account. Additionally, we lack detailed information on COVID-19 infection, such as date of diagnosis and severity of the symptoms, which could have influenced the development of antibodies and their durability over time [17,31].

Moreover, our data on COVID-19 infection were mainly based on results of tests (either PCR or serological) performed when HCW experienced symptoms or reported a high-risk contact, or via ongoing regular health surveillance. This last case refers to the Munich cohort in particular, which may have caught also asymptomatic cases. Data of infection were not available. This prevented us from calculating serology levels according to time from previous infection. To be noted, another limitation is that the analyzed serologies were not necessarily collected from the same HCW as in the previous (3-month and 6-month) analyses [15,16], limiting the interpretation of their comparison.

A further limitation is the relatively low number of subjects who received vaccines other than Comirnaty, which limited our capacity to describe different patterns of antibody levels according to vaccine type, including combinations.

This is the largest analysis on 9-month serology levels after the first dose of a COVID-19 vaccine. Positive antibodies were detected in 98.9% of the participants, and their level was directly related to female sex, previous COVID-19 infection, two vaccination doses, use of Spikevax, vaccine and heterologous vaccination. At any rate, antibody levels did not undergo a substantial decline in the previous months and at ninth month. These findings are consistent with current literature and confirm those presented in our previous analyses [15,16]. While we accounted for a number of sociodemographic and vaccine-related factors, there may be additional factors related to the development and persistence of vaccine-induced COVID-19 immunity, including host-related (such as genetics and lifestyle), vaccine-related, virus-related and environmental ones [25]. Despite the large amount of attention to COVID-19 infection, these still remain to be accurately explored.

The combination of results we presented may be interpreted as follows: in the different months (see previous ORCHESTRA analyses, [15,16]), several determinants of higher serology could be identified, also reported in the general literature; at any rate, the quantitative relation needs to be read in light of a persistent level of antibodies (see the Bologna analysis), and thus the determinants remain the same and the antibodies do not decline. This is also supported by current literature [22,23]. In this paper, we offer solid data to support the fact that vaccine-induced antibodies persist with small modification within 9 months. The next ORCHESTRA analyses will further develop this topic, expanding the current analysis and completing the Bologna one with other centers.

The data we present improve the knowledge on serological response to COVID-19 vaccines, contributing to the understanding of vaccine-induced immunity durability and effectiveness. This may in turn help to better schedule vaccination timing and administration of booster doses. Additionally, the deepening of the investigation on COVID-19 vaccines’ immunogenicity may bring new understanding regarding BI. Most importantly, in light of the expanding knowledge on COVID-19 immunization kinetics, COVID-19 vaccination should become more personalized, in order to optimize health sources and avoid unnecessary risks. Long-term observation from large multicentric prospective studies is warranted to provide more definitive evidence.

## Figures and Tables

**Table 1:** Distribution of standardized serology test results by selected characteristics.

Variables	Observed	Percent	Mean	Std. Error
**Cohort**				
Italy—Bologna	4.402	21.5	6.46	0.02
Italy—Brescia	6.250	30.5	5.79	0.01
Italy—Trieste	1995	9.7	5.83	0.03
Italy—Verona	3250	15.9	6.23	0.02
Germany—Munich	3473	17.0	2.48	0.01
Slovakia—Multicenter	567	2.8	5.75	0.05
Spain—Northern Barcelona region	412	2.0	5.61	0.06
Spain—Oviedo	127	0.6	5.20	0.16
**Gender**	3473			
Male	5515	27.2	5.41	0.02
Female	14,750	72.8	5.35	0.02
**Age group**				
≤29	2667	13.0	5.53	0.03
30–39	4555	22.2	5.42	0.02
40–49	4825	23.6	5.36	0.02
≥50	8429	41.2	5.30	0.02
**Job title**				
Administration	1364	8.1	5.75	0.03
Technician	1349	8.0	5.97	0.03
Nurses	6314	37.7	6.02	0.01
Physician (including residents)	4363	26.0	6.04	0.01
Other HCW (including auxiliary workers)	3376	20.1	5.93	0.02
**Previous COVID-19 infection** **(PCR)**				
Never infected	17,889	87.4	5.21	0.01
Infected before vaccination	2069	10.1	6.55	0.03
Infected after 1st dose	462	2.3	6.31	0.07
Infected at both times	48	0.2	6.07	0.11
**Previous COVID-19 infection** (anti-N ser. test) *				
Never infected	8313	82.1	4.17	0.02
Infected at least once	1808	17.9	6.43	0.03
**Type of vaccine**				
Comirnaty	19,824	97.1	5.39	0.01
Spikevax	446	2.2	4.91	0.10
Vaxzevria	47	0.2	3.21	0.31
Mixed vaccines	108	0.5	3.50	0.15
**Number of doses**				
One dose received	260	1.3	5.82	0.12
Two doses received	20,216	98.7	5.36	0.01

* Available for 10,121 subjects, including Brescia (*n* = 6250), Munich (*n* = 3459) and Barcelona (*n* = 412).

**Table 2:** Quantitative summary of days since previous serology test to 9-month serology test, days since 1st vaccine dose to 9-month serology test and days since last vaccine dose to 9-month serology test.

Variables	Obs	Mean	Std. dev	Minimum	Maximum
Days since previous serology test to 9-month serology test	14,541	141	43.48	1	277
Days since 1st vaccine dose to 9-month serology test	20,476	250	25.60	210	330
Days since last vaccine dose to 9-month serology test	20,476	227	28.14	0	331

**Table 3:** Relative risk of an increase of one SD in standardized log anti-S level—results of multivariate analysis.

	RR	95% CI
**Cohorts**		
Italy—Bologna	ref	
Italy—Brescia	0.42	0.41–0.44
Italy—Trieste	0.59	0.57–0.62
Italy—Verona	0.72	0.69–0.75
Germany—Munich	0.02	0.02–0.02
Slovakia—Multicentre	0.40	0.37–0.43
Spain—Northern Barcelona region	0.21	0.19–0.23
Spain—Oviedo	0.31	0.27–0.36
**Sex**		
Male	ref	
Female	1.05	1.03–1.08
**Age group**		
10 years increase	0.87	0.86–0.88
<29	-	
30–39	-	
40–49	-	
≥50	-	
**Days since last vaccine dose to 9-month serology**		
10 days increase	0.97	0.97–0.98
**Previous COVID-19 infection (detection: PCR/antiN serology test)**		
Never infected	ref	
Infected at least once	3.03	2.92–3.13
**Number of doses**		
1 dose received	ref	
2 doses received	1.22	1.09–1.36
**Type of vaccine received**		
Pfizer	ref	
Moderna	1.51	1.39–1.64
AstraZeneca	0.57	0.44–0.73
Mixed vaccines	1.33	1.12–1.57
**Previous COVID-19 infection (detection: PCR)**		
Never infected	ref	
Infected before vaccination	2.64	2.53–2.76
Infected after 1st dose	2.68	2.47–2.92
Infected at both times	2.87	2.19–3.77
Previous COVID-19 infection (detection: antiN) *		
Never infected	ref	
Infected at least once	4.02	3.86–4.19

RR, relative risk, adjusted by cohort, sex, age group, days since last vaccine dose to 9-month serology, number of doses and type of vaccine received; CI, confidence interval; ref, reference category; * available for 10,105 HCW.

## Data Availability

The datasets generated during the current study can be made available in de-identified format upon reasonable request to the principal investigators of the participating cohorts.

## Supplementary Materials

The following supporting information can be downloaded at: https://www.mdpi.com/article/10.3390/v14122657/s1, Table S1: Mean of crude serology test results, stratified by cohort; Table S2: Relative risk of an increase of one SD in standardized log anti-S level–results of multivariate analysis including job title; Table S3: Relative risk of an increase of one SD in standardized log anti-S level, stratified by type of serologic assay.
