# Supplementary material for: Determinants of Anti-S Immune Response at 9 Months after COVID-19 Vaccination in a Multicentric European Cohort of Healthcare Workers—ORCHESTRA Project
Source: Viruses. 2022 Nov 28;14(12):2657. doi: 10.3390/v14122657 (PMC9781450; doi:10.3390/v14122657)
Supplement: Supplementary file 1 [file viruses-14-02657-s001.zip › viruses-2021513 supplementary.pdf]

Supplementary Table 1. Mean of crude serology test results, stratified by cohort

Cohorts	Crude result: Mean (SE)*	Assay, sampling type
Italy-Bologna	979.06 (19.29)	Ab anti SARS CoV-2 S (RBD) IgG ECLIA
Italy-Brescia	1670.41 (27.97)	ECLIA Elecsys® anti SARS CoV2 S for anti-SARS-CoV-2-S total antibody detection (Roche Diagnostics International Ltd, Rotkreuz, Switzerland)
Italy-Trieste	2394.19 (119.88)	CMIA anti S1-RBD
Italy-Verona	745.12 (31.05)	CLIA trimeric S IgG
Germany-Munich	52.31 (2.95)‡	Roche Elecsys Anti-SARS-CoV-2 anti S1 (Roche, Mannheim, Germany)
Slovakia-Multicentre	504.46 (27.18)	QuantiVac ELISA (IgG) EUROIMMUN
Spain-Northern Barcelona region	1685.16 (122.48)	DECOV1901 ELISA (IgG-S)
Spain-Oviedo	606.96 (55.64)	COVID 19 IGG CLIA

Notes: All the serology samples were obtained from venous blood, except from Munich cohort, where samples were collected from capillary blood.

* Results adjusted by age.

‡Sample obtained from capillary blood.

Supplementary Table 2: Relative risk of an increase of one SD in standardized log anti-S level – results of multivariate analysis including job title

Variables	RR	95% CI
**Cohorts**		
Italy-Bologna	ref	
Italy-Brescia	0.43	0.42-0.45
Italy-Trieste	0.61	0.57-0.64
Italy-Verona	0.74	0.70-0.78
Slovakia-Multicentre	0.40	0.37-0.44
Spain-Barcelona	0.21	0.19-0.23
Spain-Oviedo	0.30	0.25-0.35
**Sex**		
Male	ref	
Female	1.07	1.03-1.10
**Age group**		
10 years increase	0.88	0.87-0.89
**Job title**		
Administration	ref	
Technician	1.05	0.98-1.12
Nurse	0.99	0.94-1.05
Physician (Including residents)	1.03	0.97-1.08
Other HCW (including auxiliary workers)	0.96	0.91-1.02
**Days since last vaccine dose to 9-month serology**		
10 days increase	0.98	0.97-0.99
**Previous Covid-19 infection (detection: PCR*)***		
Never infected	ref	
Infected at least once	2.96	2.86-3.07
**Number of doses**		
1 dose received	ref	
2 doses received	1.25	1.10-1.42
**Type of vaccine received**		
Pfizer	ref	
Moderna	1.72	1.53-1.93
AstraZeneca	0.64	0.40-1.04
Mixed vaccines	1.48	0.93-2.36

RR, relative risk, adjusted by cohort, sex, age group, days since last vaccine dose to 9-month serology, number of doses, type of vaccine received; CI, confidence interval; ref, reference category

Supplementary Table 3. Relative risk of an increase of one SD in standardized log anti-S level, stratified by type of serologic assay

	RBD-based CLIA (N=9,623)		Other CLIA/ELISA (N=10,593)	
**Variables**	**RR**	**95% CI**	**RR**	**95% CI**
**Sex**				
Male	ref		ref	
Female	1.05	1.01-1.09	1.07	1.03-1.11
**Age group**	**-**		ref	
10 years increase	0.87	0.85-0.88	0.87	0.86-0.88
**Days since last vaccine dose to 9-month serology**	**-**		-	
10 days increase	0.98	0.97-0.98	0.96	0.95-0.97
**Previous Covid-19 infection (detection: PCR/ antiN serology test)**				
Never infected	ref		ref	
Infected at least once	2.68	2.52-2.86	3.19	3.06-3.32
**Number of doses**				
1 dose received	ref		ref	
2 doses received	1.09	0.91-1.32	1.24	1.08-1.42
**Type of vaccine received**				
Pfizer	ref		ref	
Moderna	1.55	1.42-1.70	1.32	1.07-1.64
AstraZeneca	0.54	0.41-0.72	0.71	0.39-1.28
Mixed vaccines	1.32	1.10-1.60	1.17	0.78-1.77

RR, relative risk, adjusted by cohort, sex, age group, days since last vaccine dose to 9-month serology, number of doses, type of vaccine received; CI, confidence interval; ref, reference category
